# A Polygenic Risk Score Predicts Incident Prostate Cancer Risk in Older Men but Does Not Select for Clinically Significant Disease

**DOI:** 10.3390/cancers13225815

**Published:** 2021-11-19

**Authors:** Andrew Bakshi, Moeen Riaz, Suzanne G. Orchard, Prudence R. Carr, Amit D. Joshi, Yin Cao, Richard Rebello, Tú Nguyen-Dumont, Melissa C. Southey, Jeremy L. Millar, Lucy Gately, Peter Gibbs, Leslie G. Ford, Howard L. Parnes, Andrew T. Chan, John J. McNeil, Paul Lacaze

**Affiliations:** 1 Department of Epidemiology and Preventive Medicine, School of Public Health and Preventive Medicine, Monash University, Melbourne, VIC 3004, Australia; Moeen.Riaz@monash.edu (M.R.); suzanne.orchard@monash.edu (S.G.O.); prue.carr@monash.edu (P.R.C.); jeremy.millar@monash.edu (J.L.M.); john.mcneil@monash.edu (J.J.M.); paul.lacaze@monash.edu (P.L.); 2 Clinical and Translational Epidemiology Unit, MGH Cancer Center, Massachusetts General Hospital and Harvard Medical School, Boston, MA 02108, USA; adjoshi@mgh.harvard.edu (A.D.J.); achan@partners.org (A.T.C.); 3 Alvin J. Siteman Cancer Center, Division of Public Health Sciences, Department of Surgery, Washington University School of Medicine, St. Louis, MO 63110, USA; yin.cao@wustl.edu; 4 Centre for Cancer Research, Department of Clinical Pathology, University of Melbourne, Melbourne, VIC 3010, Australia; richard.rebello@unimelb.edu.au; 5 Sir Peter MacCallum Department of Oncology, University of Melbourne, Melbourne, VIC 3010, Australia; 6 Precision Medicine, School of Clinical Sciences at Monash Health, Monash University, Melbourne, VIC 3168, Australia; tu.nguyen-dumont@monash.edu (T.N.-D.); msouthey@unimelb.edu.au (M.C.S.); 7 Department of Clinical Pathology, University of Melbourne, Melbourne, VIC 3010, Australia; 8 Cancer Epidemiology Division, Cancer Council Victoria, Melbourne, VIC 3004, Australia; 9 Alfred Health Radiation Oncology, Alfred Hospital, Melbourne, VIC 3004, Australia; 10 Central Clinical School, Monash University, Melbourne, VIC 3168, Australia; 11 Personalised Oncology Division, Walter and Eliza Hall Institute Medical Research, Faculty of Medicine, University of Melbourne, Melbourne, VIC 3052, Australia; lucyjgately@hotmail.com (L.G.); peter.gibbs@mh.org.au (P.G.); 12 Division of Cancer Prevention, National Cancer Institute, Rockville, MD 20892, USA; fordl@mail.nih.gov (L.G.F.); parnesh@mail.nih.gov (H.L.P.)

**Keywords:** prostate cancer, polygenic risk score, Gleason grade

## Abstract

**Simple Summary:**

Prostate cancer is a common disease in older men, yet current risk prediction models for prostate cancer do not discriminate between men at higher risk of aggressive, clinically significant disease compared to those with benign disease. If risk prediction models can be improved to identify the risk of clinically significant disease with more precision, it could help to reduce over-diagnosis, over-screening, and unnecessary invasive procedures, especially in older men. We examined whether the use of a genomic risk score improves the precision and discriminative ability of prostate cancer risk prediction in men aged 70 years and older. In a population of 5701 healthy older men, we found that the genomic risk score was strongly associated with future prostate cancer risk. However, we observed no association between the genomic risk score and more aggressive, clinically significant prostate cancer—thereby limiting the clinical utility of the genomic risk score.

**Abstract:**

Despite the high prevalence of prostate cancer in older men, the predictive value of a polygenic risk score (PRS) remains uncertain in men aged ≥70 years. We used a 6.6 million-variant PRS to predict the risk of incident prostate cancer in a prospective study of 5701 men of European descent aged ≥70 years (mean age 75 years) enrolled in the ASPirin in Reducing Events in the Elderly (ASPREE) clinical trial. The study endpoint was prostate cancer, including metastatic or non-metastatic disease, confirmed by an expert panel. After excluding participants with a history of prostate cancer at enrolment, we used a multivariable Cox proportional hazards model to assess the association between the PRS and incident prostate cancer risk, adjusting for covariates. Additionally, we examined the distribution of Gleason grade groups by PRS group to determine if a higher PRS was associated with higher grade disease. We tested for interaction between the PRS and aspirin treatment. Logistic regression was used to independently assess the association of the PRS with prevalent (pre-trial) prostate cancer, reported in medical histories. During a median follow-up time of 4.6 years, 218 of the 5701 participants (3.8%) were diagnosed with prostate cancer. The PRS predicted incident risk with a hazard ratio (HR) of 1.52 per standard deviation (SD) (95% confidence interval (CI) 1.33–1.74, *p* < 0.001). Men in the top quintile of the PRS distribution had an almost three times higher risk of prostate cancer than men in the lowest quintile (HR = 2.99 (95% CI 1.90–4.27), *p* < 0.001). However, a higher PRS was not associated with a higher Gleason grade groups. We found no interaction between aspirin treatment and the PRS for prostate cancer risk. The PRS was also associated with prevalent prostate cancer (odds ratio = 1.80 per SD (95% CI 1.65–1.96), *p* < 0.001).While a PRS for prostate cancer is strongly associated with incident risk in men aged ≥70 years, the clinical utility of the PRS as a biomarker is currently limited by its inability to select for clinically significant disease.

## 1. Introduction

Prostate cancer is one of the most common cancers diagnosed in men [1,2]. Although often remaining indolent and not requiring active therapy, in some cases, prostate cancers become aggressive, leading to metastatic and incurable disease [3]—despite local or systemic treatment. Screening for prostate cancer is typically offered to men aged 50 to 69 years, using the prostate specific antigen (PSA) blood test and digital rectal examination (DRE) [4]. However, screening in older men aged ≥70 years is not recommended by contemporary guidelines [5,6], despite a high proportion of diagnoses occurring in this older age group. Some studies have suggested that targeted screening in older men could be guided by individual risk assessment [7,8]. However, current risk prediction models for prostate cancer do not discriminate between men at higher risk of aggressive, clinically significant disease, compared to those with indolent or benign disease.

If improved risk prediction models can be developed for prostate cancer with specificity for identifying risk of clinically significant disease, in turn, it could help reduce over-diagnosis, over-screening, and unnecessary invasive procedures, especially in older men [9,10], by focusing on early detection and prevention strategies for men at highest risk. Improved risk prediction models could also help direct the use of emerging non-invasive screening methods, such as magnetic resonance imaging (MRI) [11].

Although modifiable risk factors (such as smoking) are associated with prostate cancer risk [12,13,14], there is limited evidence that prevention is achieved by the cessation of these factors [15,16]. The most significant risk factors for prostate cancer are non-modifiable, such as age, ethnicity [17], family history [18], and genetic predisposition [19]. Therefore, the use of a polygenic risk score (PRS) comprised of common prostate cancer-associated genetic variants has the potential to improve risk prediction, beyond conventional risk factors. Genome-wide association and twin studies have estimated the heritability of prostate cancer to be 57% [20,21]. Whilst a small proportion of this heritability is explained by rare pathogenic variants in monogenic disease-associated genes [22,23,24,25], a larger proportion is attributable to common variants identified by genome-wide association studies (GWAS). A PRS aggregates the effect of these common variants into a single measure of genetic risk that can then be used alongside conventional prostate cancer risk factors in a risk prediction model. Polygenic scores for prostate cancer have been shown to be strongly associated with the development of prostate cancer in previous studies [26,27,28,29], including in diverse genetic ancestries [30]. In these studies, the SNP selection has been predominantly limited to tens to hundreds of genome-wide significant variants.

A recent study developed a 6.6 million variant PRS for prostate cancer risk based on a recent GWAS [20,26,27]. We sought to validate the performance of this PRS independently in a well-characterised prospective cohort of older men from Australia and the United States of America aged ≥70 years. The goal of our study is to determine whether the PRS predicts incident prostate cancer risk in older men, and test whether the PRS has the discriminative ability to identify risk of clinically significant disease (as determined by Gleason grade group [28]), to help guide targeted screening in older men.

## 2. Methods

### 2.1. Study Design

This study involved a post hoc genetic analysis of all male participants of non-Finnish European ancestry from the ASPirin in Reducing Events in the Elderly (ASPREE) trial—a randomised placebo-controlled trial of daily low-dose aspirin in older people [29,30,31]. The design [32,33], recruitment [34], and baseline characteristics [35] of the ASPREE study have been published previously. Of the 6046 male participants in ASPREE who provided DNA samples and consent for genetic analysis, 318 participants of non-European ancestry were removed from the analysis using principal component analysis (PCA)-based filtering (see Supplementary Materials, Table S1), while a further 27 were removed due to missing covariates, resulting in a total of 5701 male participants. All participants were aged 70 years or older at enrolment with genotyping data available [36,37] and provided informed consent for genetic research at the time of sample collection. The ASPREE study is registered on Clinicaltrials.gov (NCT01038583) and approved by local ethics committees.

### 2.2. Genotyping

Genotyping was performed on the Axiom 2.0 Precision Medicine Diversity Research Array (Thermo Fisher Scientific (TFS), Waltham, MA, USA) following standard protocols. Variants were aligned to the human genome reference GRCh38. Participants with European ancestry were included to minimise the effect of population stratification on polygenic risk scoring. PCA was used to identify ethnicity and filter participants not overlapping with the non-Finnish European subset of the 1000 genomes reference population (Supplementary Figure S1) [38]. Imputation was performed on the TopMED Imputation Server (European samples) [39]. Variants with low imputation quality scores (r^2^ < 0.3) were removed, as were multi-allelic variants resulting in 98.5% of SNPs (single-nucleotide polymorphisms) (6,508,423 of the 6,606,786) in the PRS passing quality control.

### 2.3. Endpoint

The study endpoint was prostate cancer diagnosis, as adjudicated by an expert panel, with histopathology or imaging of metastasis to confirm diagnosis as described previously [40]. Incident cases included both localised and metastatic prostate cancer. Metastatic recurrence was reported but excluded from the analysis of incident prostate cancer events. The median follow-up time among male participants was 4.6 years. Prostate cancer occurring prior to enrolment was self-reported (hereon referred to as prevalent cancer), with age at diagnosis listed as either occurring at 49 years or younger, or at age 50 or older. The analysis of incident prostate cancer risk excluded all participants with prevalent prostate cancer at enrolment.

### 2.4. Calculation of Polygenic Risk Score

The PRS was calculated using the ‘score’ function from plink version 1.9 [41,42]. The PRS is defined as the sum of all the effect sizes for each effect allele present in a participant, taken from the published GWAS summary statistics [20]. The PRS was developed by Mars et al. (2020) [26] who used LDpred to prune and select 6,606,785 SNPs following methods described previously. The list of SNPs and effect sizes were downloaded from the polygenic score (PGS) catalog [43].

### 2.5. Statistical Analysis

The association between the PRS and incident prostate cancer was evaluated using a multivariable Cox proportional hazards model, excluding all participants with prevalent prostate cancer at enrolment. Hazard ratios (HRs) were reported, adjusting for age, first degree family history of prostate cancer, and randomization to aspirin treatment. The PRS was also assessed in an independent model as a categorical variable, categorised into low (Q1: 0–20%), medium (Q2–Q4: >20–80%), and high (Q5: >80%) risk groups. The c-index (concordance index) was used to assess the discriminative ability of the models. We used a chi-squared test to examine the distribution of Gleason grade groups across categorical PRS risk groups. We tested whether the PRS and aspirin treatment had an interaction effect on the risk of incident prostate cancer risk using the Wald test, and adjusting for the same covariates. Competing risks (from death) estimates of the cumulative incidence were visualised using the survfit function from the R survival [44] and survminer 0.4.8 packages. Statistical analyses were conducted using R v3.6.1 [45] with tidyverse 1.3.0 [46].

Prevalent prostate cancer at enrolment was analysed using a logistic regression model, to report the odds ratio (OR) of the PRS. The prevalence model also included the covariates of age, and family history of prostate cancer.

## 3. Results

### 3.1. Baseline Characteristics

The mean age of participants at enrolment was 75 years, with more than half of the participants aged between 70 and 74 years at enrolment (Table 1). Of 5701 male participants, 9% reported a family history of prostate cancer, 56% were current or former smokers, and 11% reported prior diagnosis of prostate cancer at the time of enrolment. The PRS was normally distributed with a mean value of −0.46 (SD 0.16). In subsequent analysis, this was standardised to have mean 0 (SD 1).

### 3.2. Prostate Cancer Diagnoses

During a median follow-up time of 4.6 years, 218 participants out of 5701 (3.8%) were diagnosed with prostate cancer. Of these, 31 diagnoses were for metastatic prostate cancer. The majority of diagnoses occurred in participants aged 70–74 years and were identified as stage II disease (organ confined) (Supplementary Figure S2).

### 3.3. Association of PRS with Incident Prostate Cancer Risk

After excluding participants with prevalent prostate cancer at baseline, the PRS as a continuous variable was associated with increased incident risk of prostate cancer with a hazard ratio (HR) of 1.52 per SD of the PRS (95% CI 1.33–1.74, *p <* 0.001), after adjustment for relevant covariates (Table 2). When considering the model without the PRS, the c-index was 0.52 (95% CI 0.48–0.55). After addition of the PRS, the c-index improved to 0.62 (95% CI 0.58; 0.66). We tested for an interaction between the PRS and aspirin treatment, but found no interaction (*p* > 0.05).

**Figure 1:**
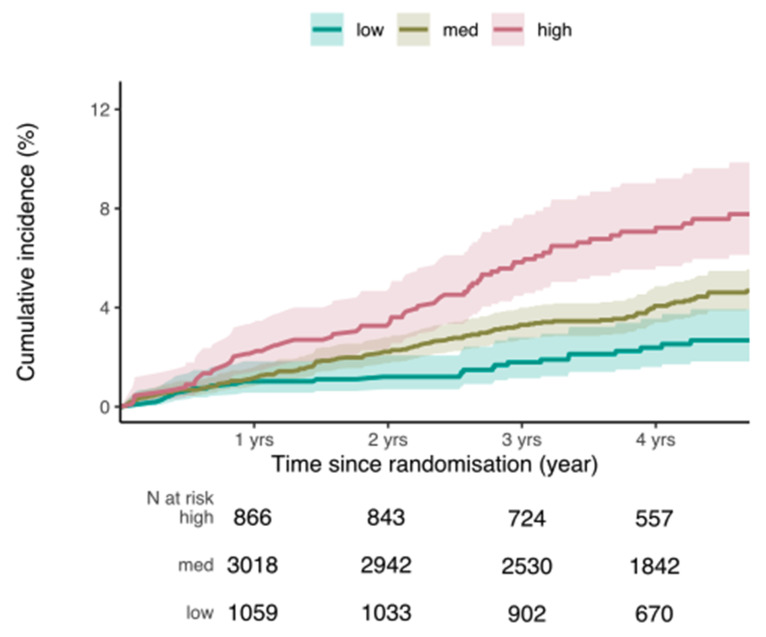
Competing risk survival curves for incident prostate cancer by PRS group. Low risk = PRS 0–20% (blue line); medium risk = PRS > 20–80% (yellow line); high risk = PRS > 80–100% (red line). Cumulative incidence of prostate cancer risk accounting for competing risk (death) is plotted, with groups stratified according to PRS group.

Compared with participants in the lowest quintile of the PRS distribution (Q1), participants in the medium-risk PRS group (Q2–4) had a higher risk of prostate cancer (Figure 1), with an HR of 1.74 (95% CI 1.14; 2.66, *p* = 0.01). Participants in the highest quintile of polygenic risk (Q5) had an almost three times higher risk of incident prostate cancer than participants in the lowest PRS quintile, with an HR of 2.99 (95% CI 1.90; 4.72, *p* < 0.001) (Table 2).

Only 20 of the 218 participants (9.2%) with an incident prostate cancer diagnosis reported a family history of prostate cancer at enrolment. When excluding these participants from the analysis, the PRS remained associated with incident prostate cancer risk (HR = 1.50 per SD, 95% CI 1.30–1.72, *p* < 0.001).

In subgroup analysis, when only participants aged 70–74 years at enrolment were analysed, the HR of the PRS was 1.65 per SD (95% CI 1.39–1.97), with the high-risk PRS group having over three times higher risk than the low-risk PRS group (Q5 vs. Q1 HR = 3.54 (95% CI 1.93–6.49)) (Table 3).

**Figure 2:**
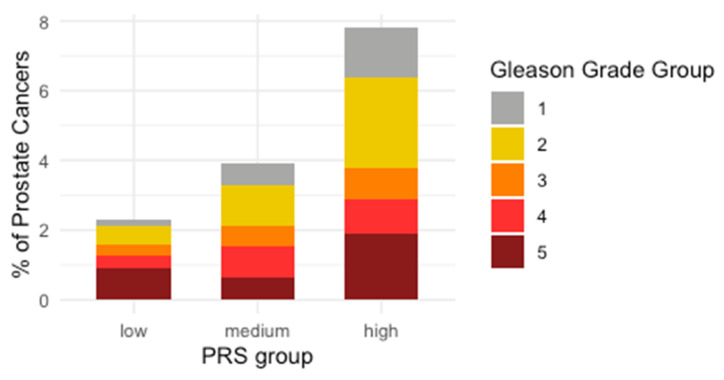
Gleason grade in incident prostate cancer stratified by polygenic risk, showing percentage of prostate cancers in each group, divided by their Gleason grade group. Gleason scores were retrieved from 191 individuals. Scores from the primary and secondary foci were combined together to report the Gleason grade group (Table 2, Figure 1). Gleason grade group 2 was the most commonly diagnosed across the cohort. There was no evidence of enrichment of Gleason grade groups across PRS categories (chisq = 8.5, df = 8, *p* = 0.37).

The distribution of incident prostate cancer diagnoses by the Gleason grade group [28] (GrG) was assessed using chi-squared tests (Figure 2). The analysis found that the Gleason GrGs were distributed evenly among the PRS distribution with no statistically significant evidence of enrichment in any PRS group (chisq = 8.5, df = 8, *p* = 0.37) and no association of the PRS with higher-grade disease. Despite observing a large proportion of GrG 5 tumours in the low PRS group, there was no statistically significant difference in the distribution of GrGs when examining only this grade group (chisq = 5.6, df = 2, *p* = 0.06). We also examined staging information, where data were available (in 211 cases), stratifying the data into three groups—I, II/III, IV). We found no evidence of enrichment of stage in any PRS group (chisq = 3.68, df = 4, *p* = 0.45).

### 3.4. Prevalent Prostate Cancer

A total of 658 participants reported a prior history of prostate cancer at enrolment (prevalent prostate cancer), of which 654 were diagnosed at age 50 or older, while only four occurred at age 49 years or younger. The PRS as a continuous variable was associated with prevalent prostate cancer (OR = 1.80 per SD, 95% CI 1.65–1.96, *p* < 0.001). When categorized into low (reference), medium, and high PRS groups, the medium group was associated with increased risk of prevalent prostate cancer (OR = 1.93, 95% CI 1.47–2.57) compared to the low PRS group, and the high PRS group (highest quintile) was associated with over four times higher risk (OR= 4.6, 95% CI 3.48–6.26, *p* < 0.001) compared to the low PRS group.

## 4. Discussion

We assessed the predictive value of a recently developed PRS for the prediction of incident prostate cancer risk in older men aged ≥70 years. Overall, the PRS was a strong predictor of risk, with an HR of 1.52 per SD during a median 4.6 years of follow-up. Men in the highest quintile of the PRS distribution were almost three times more likely to develop incident prostate cancer than men in the lowest quintile during this period (HR = 2.99). However, notably, the PRS was not able to identify men at risk of clinically significant prostate cancer (as defined by a higher Gleason grade group). This major limitation of the PRS currently restricts its clinical utility for risk prediction in older men, and for guiding targeted screening. Unless improved specificity can be achieved through future refinement or iteration of the current PRS, or by combining it with other biomarkers or screening modalities, the inability to discriminate between clinically significant and clinically insignificant disease will likely limit the clinical application of the PRS, at least in the context of primary prevention and routine screening.

Despite this limitation, the observed predictive ability of the prostate cancer PRS in the ASPREE trial population was higher than most other PRSs we have tested for other conditions [36,37,47,48]. This includes risk prediction studies of PRSs for melanoma [37], breast cancer [47], coronary artery disease [49], and ischemic stroke [48], where HRs of the PRSs have been lower in the same trial population. The stronger effect (HR) of the prostate cancer PRS could reflect the high heritability of the disease, and the significant role of genetic predisposition as a risk factor. In addition to the observed strong effect of the PRS in predicting incident prostate cancer risk in older men, we also found a strong association between the PRS and prevalent prostate cancer (in men who reported a personal history of disease prior to enrolment). The association of the PRS with prevalent prostate cancer (OR = 1.80) was similar to the HR of 1.83 reported in the original study of younger men from which the PRS was derived [26]. This shows consistent performance of the PRS when assessed in an independent study of men of European-descent.

However, despite the predictive performance of the PRS for identifying the risk of incident prostate cancer in the ASPREE population, we observed no evidence of the PRS being capable of discriminating between men at risk of high-grade versus low-grade prostate cancer. This result was consistent with other studies in different populations [50,51,52,53]. This lack of association with tumor grade limits the potential clinical utility of the PRS, and has implications for the consideration of genomic risk prediction in targeted screening for prostate cancer. A further limitation of the PRS is that this measure remains constant and unchanged throughout an individual’s lifetime and is not yet an established clinical test. By contrast, current PSA testing has the advantage of being a dynamic marker which can change during disease progression, with a higher PSA indicating a greater likelihood of risk, and it can be measured using an established low-cost laboratory test.

Furthermore, the lack of specificity of the current PRS to identify the risk of aggressive disease can likely be linked back to the initial ascertainment of prostate cancer cases used in the original GWAS to derive the score [26]. The original GWAS reported associations based on low- and high-grade prostate cancer cases combined. As the PRS was derived from analysis irrespective of tumor grade, it may not be surprising that it lacks the specificity to distinguish clinically-significant from clinically-insignificant disease in independent study populations.

One possible way to overcome this limitation would be to conduct a large GWAS using only high-grade and aggressive prostate cancer cases to derive a new high-grade specific PRS. However, in the original GWAS from which the current PRS was derived [20], the authors did attempt to stratify individuals by low-versus high-grade as well as age of onset, and identified only one variant (rs138004030) associated with early onset disease, and no strong evidence of genetic associations with aggressive or advanced disease [20].

An alternative approach might be to stratify based on genomic features, rather than clinical grade. In a recent pan-cancer study, common germline variants were shown to explain only a proportion of variation of tumour mutational burden across cancers [54]—a feature typically associated with unique molecular features such as response to immunotherapy [55]. In prostate cancer, high levels of tumour copy number burden are associated with the development of biochemical recurrence and treatment failure [56,57]. Therefore, it could be hypothesized that a PRS based on common variants that are associated with higher copy number burden could explain a greater proportion of genetic risk for aggressive disease. If so, developing and adding these additional genomic measures to a combined risk score (with the PRS) could offer additional pathways to improve the specificity of risk prediction for aggressive disease.

Rare high-penetrance pathogenic variants can also contribute to the risk of aggressive prostate cancer, including those in known susceptibility genes such as *BRCA2* [58]. However, these are only found in a small number of high-risk men in the population (<1% of males in the general population [25]), meaning monogenic testing alone is not likely to be tractable for guiding targeted screening. In the future, however, the concept of a combined genomic risk score that incorporates common, rare, and structural variants for prostate cancer predisposition may help improve the specificity and sensitivity for predicting aggressive prostate cancer risk, beyond the current PRS.

Strengths of our study include a well-characterised prospective cohort of older men, with adjudicated pathological assessment of prostate cancer cases. The ASPREE cohort represents an older demographic, where a high proportion of prostate cancer diagnoses occur, who have historically been under-represented in genomic risk prediction studies. The ASPREE cohort was selected for men without serious or chronic illness that would impact survival over the course of the trial, reflecting a key demographic of largely healthy individuals who are more likely to consider screening, with fewer competing risks and comorbidities than the general population.

The limitations of the study include the lack of data on prostate cancer screening history, as well as a lack of access to baseline and ongoing PSA measurements to assess the overlap between risk estimates derived from a high PRS or a high PSA test. Our study is also limited in a lack of genetic diversity, resulting in an inability to generalise results to other genetic ancestries or diverse populations [50].

The goal of improving prostate cancer risk prediction towards the identification of clinically significant disease remains a challenge. However, if further progress can be made in the refinement of current genomic risk scores and other complementary measures, then improved risk prediction could help reduce over-diagnosis, over-screening, and unnecessary invasive procedures, especially in older men [4,10]. While the current PRS is strongly associated with incident prostate cancer risk in older men overall, the clinical utility of this biomarker is currently limited by its inability to discriminate clinically significant from clinically insignificant disease.

## Figures and Tables

**Table 1:** Baseline characteristics of the included ASPREE male participants.

Characteristics	ASPREE
Participants	5701
Age at randomisation (mean (SD))	74.9 (4.2)
Age Group (%)	
70–74	3566 (62.6)
75–79	1384 (24.3)
80–84	585 (10.3)
85+	166 (2.9)
Current or former smoker (%)	3203 (56.2)
Diabetes (%)	637 (11.2)
Randomized to Aspirin (%)	2837 (49.8)
Body-mass-index (kg/m^2^) − mean (SD)	27.9 (3.8)
Current alcohol consumption (%)	4880 (85.6)
Family history of prostate cancer (%)	499 (8.8)
Polygenic Risk Score − mean (SD)	−0.46 (0.15)
Prevalent Prostate Cancer (%) (self-reported at enrolment)	
None	4725 (88.5)
<49 years	4 (0.0)
≥50 years	654 (11.5)

**Table 2:** Multivariable Cox regression model for prostate cancer incidence.

**Incident prostate cancer.** (218 clinically confirmed cases during the ASPREE trial, excluding all prevalent cases)						
**Variable**	**PRS as Continuous Variable** **(per Standard Deviation)**			**PRS as Categorical Variable** **(low, medium, high)**		
	**Hazard Ratio**	**95% CI**	***p*-Value**	**Hazard Ratio**	**95% CI**	***p*-Value**
**PRS** (per std dev)	1.52	(1.33; 1.74)	<0.0001			
**Low PRS** 0–20% (*n* = 23)				Reference		
**Medium PRS** >20–80% (*n* = 128)				1.74	(1.14; 2.66)	0.01
**High PRS** >80% (*n* = 57)				2.99	(1.90; 4.72)	<0.0001

Cox proportional hazards model, adjusted for first-degree family history of prostate cancer, age at enrolment, and trial arm (aspirin/placebo).

**Table 3:** Multivariable Cox regression model for prostate cancer incidence—subgroup analysis for men aged 70–74 years.

**Incident prostate cancer in participants with age at randomisation between 70 and 74 years** (126 clinically confirmed cases during the ASPREE trial, excluding all prevalent cases)						
**Variable**	**PRS as Continuous Variable** **(per Standard Deviation)**			**PRS as Categorical Variable ** **(low, medium, high)**		
	**Hazard Ratio**	**95% CI**	***p*-Value**	**Hazard Ratio**	**95% CI**	***p*-Value**
**PRS** (per std dev)	1.65	(1.39; 1.97)	<0.0001			
**Low PRS** 0–20% (*n* = 23)				Reference		
**Medium PRS** >20–80% (*n* = 128)				1.78	(1.00; 3.15)	0.05
**High PRS** >80% (*n* = 57)				3.54	(1.93; 6.49)	<0.0001

Cox proportional hazards model, adjusted for first degree family history of prostate cancer, age at enrolment, and trial arm (aspirin/placebo).

## Data Availability

The data that support the findings of this study are available from the corresponding author upon request.

## Supplementary Materials

The following are available online at https://www.mdpi.com/article/10.3390/cancers13225815/s1, Figure S1: principal component (PC) analysis of the ASPREE cohort compared with the 1000 Genome Project, Figure S2: TNM staging by age, Table S1: association of the PRS with prevalent prostate cancer.

## References

[1] Pernar C.H., Ebot E.M., Wilson K.M., Mucci L.A. (2018). The Epidemiology of Prostate Cancer. Cold Spring Harb. Perspect. Med..

[2] Bray F., Ferlay J., Soerjomataram I., Siegel R.L., Torre L.A., Jemal A. (2018). Global Cancer Statistics 2018: GLOBOCAN Estimates of Incidence and Mortality Worldwide for 36 Cancers in 185 Countries. CA Cancer J. Clin..

[3] Sartor O., de Bono J.S. (2018). Metastatic Prostate Cancer. N. Engl. J. Med..

[4] Hayes J.H., Barry M.J. (2014). Screening for Prostate Cancer with the Prostate-Specific Antigen Test: A Review of Current Evidence. JAMA.

[5] Grossman D.C., Curry S.J., Owens D.K., Bibbins-Domingo K., Caughey A.B., Davidson K.W., Doubeni C.A., Ebell M., Epling J.W., US Preventive Services Task Force (2018). Screening for Prostate Cancer: US Preventive Services Task Force Recommendation Statement. JAMA.

[6] Clinical Practice Guidelines on PSA Testing PCFA. https://www.prostate.org.au/awareness/for-healthcare-professionals/clinical-practice-guidelines-on-psa-testing/.

[7] American Cancer Society Recommendations for Prostate Cancer Early Detection. https://www.cancer.org/cancer/prostate-cancer/detection-diagnosis-staging/acs-recommendations.html.

[8] Boyle H.J., Alibhai S., Decoster L., Efstathiou E., Fizazi K., Mottet N., Oudard S., Payne H., Prentice M., Puts M. (2019). Updated Recommendations of the International Society of Geriatric Oncology on Prostate Cancer Management in Older Patients. Eur. J. Cancer.

[9] Louie K.S., Seigneurin A., Cathcart P., Sasieni P. (2015). Do Prostate Cancer Risk Models Improve the Predictive Accuracy of PSA Screening? A Meta-Analysis. Ann. Oncol..

[10] Mottet N., Bellmunt J., Bolla M., Briers E., Cumberbatch M.G., De Santis M., Fossati N., Gross T., Henry A.M., Joniau S. (2017). EAU-ESTRO-SIOG Guidelines on Prostate Cancer. Part 1: Screening, Diagnosis, and Local Treatment with Curative Intent. Eur. Urol..

[11] Stabile A., Giganti F., Rosenkrantz A.B., Taneja S.S., Villeirs G., Gill I.S., Allen C., Emberton M., Moore C.M., Kasivisvanathan V. (2020). Multiparametric MRI for Prostate Cancer Diagnosis: Current Status and Future Directions. Nat. Rev. Urol..

[12] Peisch S.F., Van Blarigan E.L., Chan J.M., Stampfer M.J., Kenfield S.A. (2017). Prostate Cancer Progression and Mortality: A Review of Diet and Lifestyle Factors. World J. Urol..

[13] Brookman-May S.D., Campi R., Henríquez J.D.S., Klatte T., Langenhuijsen J.F., Brausi M., Linares-Espinós E., Volpe A., Marszalek M., Akdogan B. (2019). Latest Evidence on the Impact of Smoking, Sports, and Sexual Activity as Modifiable Lifestyle Risk Factors for Prostate Cancer Incidence, Recurrence, and Progression: A Systematic Review of the Literature by the European Association of Urology Section of Oncological Urology (ESOU). Eur. Urol. Focus.

[14] Patel A.R., Klein E.A. (2009). Risk Factors for Prostate Cancer. Nat. Clin. Pract. Urol..

[15] Foerster B., Pozo C., Abufaraj M., Mari A., Kimura S., D’Andrea D., John H., Shariat S.F. (2018). Association of Smoking Status With Recurrence, Metastasis, and Mortality Among Patients With Localized Prostate Cancer Undergoing Prostatectomy or Radiotherapy: A Systematic Review and Meta-Analysis. JAMA Oncol..

[16] Allott E.H., Masko E.M., Freedland S.J. (2013). Obesity and Prostate Cancer: Weighing the Evidence. Eur. Urol..

[17] Cuzick J., Thorat M.A., Andriole G., Brawley O.W., Brown P.H., Culig Z., Eeles R.A., Ford L.G., Hamdy F.C., Holmberg L. (2014). Prevention and Early Detection of Prostate Cancer. Lancet Oncol..

[18] Albright F., Stephenson R.A., Agarwal N., Teerlink C.C., Lowrance W.T., Farnham J.M., Albright L.A.C. (2015). Prostate Cancer Risk Prediction Based on Complete Prostate Cancer Family History. Prostate.

[19] Doan D.K., Schmidt K.T., Chau C.H., Figg W.D. (2021). Germline Genetics of Prostate Cancer: Prevalence of Risk Variants and Clinical Implications for Disease Management. Cancers.

[20] Schumacher F.R., Al Olama A.A., Berndt S.I., Benlloch S., Ahmed M., Saunders E.J., Dadaev T., Leongamornlert D., Anokian E., Cieza-Borrella C. (2018). Association Analyses of More than 140,000 Men Identify 63 New Prostate Cancer Susceptibility Loci. Nat. Genet..

[21] Mucci L.A., Hjelmborg J.B., Harris J.R., Czene K., Havelick D.J., Scheike T., Graff R.E., Holst K., Möller S., Unger R.H. (2016). Familial Risk and Heritability of Cancer Among Twins in Nordic Countries. JAMA.

[22] Mancuso N., Rohland N., Rand K.A., Tandon A., Allen A., Quinque D., Mallick S., Li H., Stram A., Sheng X. (2016). The Contribution of Rare Variation to Prostate Cancer Heritability. Nat. Genet..

[23] Leongamornlert D., Mahmud N., Tymrakiewicz M., Saunders E., Dadaev T., Castro E., Goh C., Govindasami K., Guy M., O’Brien L. (2012). Germline BRCA1 Mutations Increase Prostate Cancer Risk. Br. J. Cancer.

[24] Karlsson R., Aly M., Clements M., Zheng L., Adolfsson J., Xu J., Grönberg H., Wiklund F. (2014). A Population-Based Assessment of Germline HOXB13 G84E Mutation and Prostate Cancer Risk. Eur. Urol..

[25] Nguyen-Dumont T., MacInnis R.J., Steen J.A., Theys D., Tsimiklis H., Hammet F., Mahmoodi M., Pope B.J., Park D.J., Mahmood K. (2020). Rare Germline Genetic Variants and Risk of Aggressive Prostate Cancer. Int. J. Cancer.

[26] Mars N., Koskela J.T., Ripatti P., Kiiskinen T.T.J., Havulinna A.S., Lindbohm J.V., Ahola-Olli A., Kurki M., Karjalainen J., Palta P. (2020). Polygenic and Clinical Risk Scores and Their Impact on Age at Onset and Prediction of Cardiometabolic Diseases and Common Cancers. Nat. Med..

[27] Vilhjálmsson B.J., Yang J., Finucane H.K., Gusev A., Lindström S., Ripke S., Genovese G., Loh P.-R., Bhatia G., Do R. (2015). Modeling Linkage Disequilibrium Increases Accuracy of Polygenic Risk Scores. Am. J. Hum. Genet..

[28] Epstein J.I., Egevad L., Amin M.B., Delahunt B., Srigley J.R., Humphrey P.A. (2016). The 2014 International Society of Urological Pathology (ISUP) Consensus Conference on Gleason Grading of Prostatic Carcinoma. Am. J. Surg. Pathol..

[29] McNeil J.J., Wolfe R., Woods R.L., Tonkin A.M., Donnan G.A., Nelson M.R., Reid C.M., Lockery J.E., Kirpach B., Storey E. (2018). Effect of Aspirin on Cardiovascular Events and Bleeding in the Healthy Elderly. N. Engl. J. Med..

[30] McNeil J.J., Woods R.L., Nelson M.R., Reid C.M., Kirpach B., Wolfe R., Storey E., Shah R.C., Lockery J.E., Tonkin A.M. (2018). Effect of Aspirin on Disability-Free Survival in the Healthy Elderly. N. Engl. J. Med..

[31] McNeil J.J., Nelson M.R., Woods R.L., Lockery J.E., Wolfe R., Reid C.M., Kirpach B., Shah R.C., Ives D.G., Storey E. (2018). Effect of Aspirin on All-Cause Mortality in the Healthy Elderly. N. Engl. J. Med..

[32] ASPREE Investigator Group (2013). Study Design of ASPirin in Reducing Events in the Elderly (ASPREE): A Randomized, Controlled Trial. Contemp. Clin. Trials.

[33] Nelson M.R., Reid C.M., Ames D., Beilin L.J., Donnan G.A., Gibbs P., Johnston C.I., Krum H., Storey E., Tonkin A. (2008). Feasibility of Conducting a Primary Prevention Trial of Low-dose Aspirin for Major Adverse Cardiovascular Events in Older People in Australia: Results from the ASPirin in Reducing Events in the Elderly (ASPREE) Pilot Study. Med. J. Aust..

[34] Lockery J.E., Collyer T.A., Abhayaratna W.P., Fitzgerald S.M., McNeil J.J., Nelson M.R., Orchard S.G., Reid C., Stocks N.P., Trevaks R.E. (2019). Recruiting General Practice Patients for Large Clinical Trials: Lessons from the Aspirin in Reducing Events in the Elderly (ASPREE) Study. Med. J. Aust..

[35] McNeil J.J., Woods R.L., Nelson M.R., Murray A.M., Reid C.M., Kirpach B., Storey E., Shah R.C., Wolfe R.S., Tonkin A.M. (2017). Baseline Characteristics of Participants in the ASPREE (ASPirin in Reducing Events in the Elderly) Study. J. Gerontol. Ser. A.

[36] Riaz M., Huq A., Ryan J., Orchard S.G., Tiller J., Lockery J., Woods R.L., Wolfe R., Renton A.E., Goate A.M. (2021). Effect of APOE and a Polygenic Risk Score on Incident Dementia and Cognitive Decline in a Healthy Older Population. Aging Cell.

[37] Bakshi A., Yan M., Riaz M., Polekhina G., Orchard S.G., Tiller J., Wolfe R., Joshi A., Cao Y., McInerney-Leo A.M. (2021). Genomic Risk Score for Melanoma in a Prospective Study of Older Individuals. J. Natl. Cancer Inst..

[38] Consortium T. (2015). 1000 G.P.; The 1000 Genomes Project Consortium A Global Reference for Human Genetic Variation. Nature.

[39] Taliun D., Harris D.N., Kessler M.D., Carlson J., Szpiech Z.A., Torres R., Taliun S.A.G., Corvelo A., Gogarten S.M., Kang H.M. (2021). Sequencing of 53,831 Diverse Genomes from the NHLBI TOPMed Program. Nature.

[40] McNeil J.J., Gibbs P., Orchard S.G., Lockery J.E., Bernstein W.B., Cao Y., Ford L., Haydon A., Kirpach B., Macrae F. (2021). Effect of Aspirin on Cancer Incidence and Mortality in Older Adults. J. Natl. Cancer Inst..

[41] Purcell S., Neale B., Todd-Brown K., Thomas L., Ferreira M.A.R., Bender D., Maller J., Sklar P., de Bakker P.I.W., Daly M.J. (2007). PLINK: A Tool Set for Whole-Genome Association and Population-Based Linkage Analyses. Am. J. Hum. Genet..

[42] Chang C.C., Chow C.C., Tellier L.C., Vattikuti S., Purcell S.M., Lee J.J. (2015). Second-Generation PLINK: Rising to the Challenge of Larger and Richer Datasets. Gigascience.

[43] Lambert S.A., Gil L., Jupp S., Ritchie S.C., Xu Y., Buniello A., McMahon A., Abraham G., Chapman M., Parkinson H. (2021). The Polygenic Score Catalog as an Open Database for Reproducibility and Systematic Evaluation. Nat. Genet..

[44] Therneau T.M., Lumley T. (2015). Package “survival”. R Top Doc.

[45] Team R.C. Others R: A Language and Environment for Statistical Computing. https://www.r-project.org/.

[46] Wickham H., Averick M., Bryan J., Chang W., McGowan L., François R., Grolemund G., Hayes A., Henry L., Hester J. (2019). Welcome to the Tidyverse. J. Open Source Softw..

[47] Lacaze P., Bakshi A., Riaz M., Orchard S.G., Tiller J., Neumann J.T., Carr P.R., Joshi A.D., Cao Y., Warner E.T. (2021). Genomic Risk Prediction for Breast Cancer in Older Women. Cancers.

[48] Neumann J.T., Riaz M., Bakshi A., Polekhina G., Thao L.T.P., Nelson M.R., Woods R.L., Abraham G., Inouye M., Reid C.M. (2021). Predictive Performance of a Polygenic Risk Score for Incident Ischemic Stroke in a Healthy Older Population. Stroke.

[49] Neumann J.T., Riaz M., Bakshi A., Polekhina G., Thao L.T.P., Nelson M.R., Woods R.L., Abraham G., Inouye M., Reid C.M. (2021). A Polygenic Risk Score for Coronary Heart Disease Performs Well in Individuals Aged 70 Years and Older. medRxiv.

[50] Plym A., Penney K.L., Kalia S., Kraft P., Conti D.V., Haiman C., Mucci L.A., Kibel A.S. (2021). Evaluation of a Multiethnic Polygenic Risk Score Model for Prostate Cancer. J. Natl. Cancer Inst..

[51] Black M.H., Li S., LaDuca H., Lo M.-T., Chen J., Hoiness R., Gutierrez S., Tippin-Davis B., Lu H.-M., Gielzak M. (2020). Validation of a Prostate Cancer Polygenic Risk Score. Prostate.

[52] Pashayan N., Duffy S.W., Neal D.E., Hamdy F.C., Donovan J.L., Martin R.M., Harrington P., Benlloch S., Amin Al Olama A., Shah M. (2015). Implications of Polygenic Risk-Stratified Screening for Prostate Cancer on Overdiagnosis. Genet. Med..

[53] Sipeky C., Talala K.M., Tammela T.L.J., Taari K., Auvinen A., Schleutker J. (2020). Prostate Cancer Risk Prediction Using a Polygenic Risk Score. Sci. Rep..

[54] Sun X., Xue A., Qi T., Chen D., Shi D., Wu Y., Zheng Z., Zeng J., Yang J. (2021). Tumor Mutational Burden Is Polygenic and Genetically Associated with Complex Traits and Diseases. Cancer Res..

[55] Sha D., Jin Z., Budczies J., Kluck K., Stenzinger A., Sinicrope F.A. (2020). Tumor Mutational Burden as a Predictive Biomarker in Solid Tumors. Cancer Discov..

[56] Hieronymus H., Schultz N., Gopalan A., Carver B.S., Chang M.T., Xiao Y., Heguy A., Huberman K., Bernstein M., Assel M. (2014). Copy Number Alteration Burden Predicts Prostate Cancer Relapse. Proc. Natl. Acad. Sci. USA.

[57] Hieronymus H., Murali R., Tin A., Yadav K., Abida W., Moller H., Berney D., Scher H., Carver B., Scardino P. (2018). Tumor Copy Number Alteration Burden Is a Pan-Cancer Prognostic Factor Associated with Recurrence and Death. Elife.

[58] Taylor R.A., Fraser M., Livingstone J., Espiritu S.M.G., Thorne H., Huang V., Lo W., Shiah Y.-J., Yamaguchi T.N., Sliwinski A. (2017). Germline BRCA2 Mutations Drive Prostate Cancers with Distinct Evolutionary Trajectories. Nat. Commun..

