# Supplementary material for: A Polygenic Risk Score Predicts Incident Prostate Cancer Risk in Older Men but Does Not Select for Clinically Significant Disease
Source: Cancers (Basel). 2021 Nov 19;13(22):5815. doi: 10.3390/cancers13225815 (PMC8616400; doi:10.3390/cancers13225815)
Supplement: Supplementary file 1 [file cancers-13-05815-s001.zip › cancers-1462280-supplementary.pdf]

cancers

MDPI

Supplementary Materials

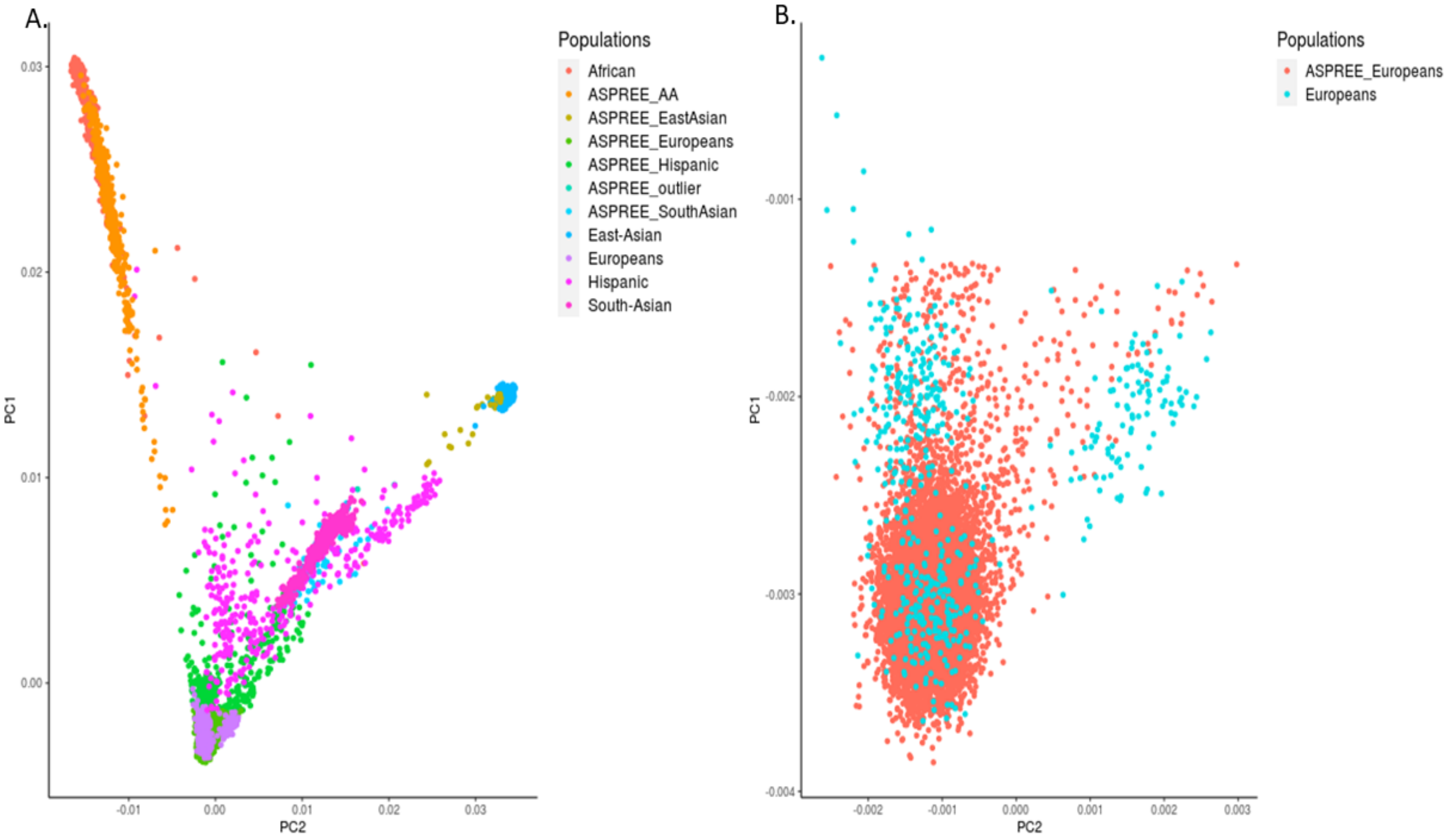

Figure S1. Principal component (PC) analysis of the ASPREE cohort compared with the 1,000 Genome Project. **(A)** PC plot of all genoptyped ASPREE participants mapped against the 1,000 Genome population groups (Europeans, South Asians, East Asians, African American and Hispanics). (ASPREE_AA = ASPREE participants of African American descent). **(B)** PC plot of European ASPREE genotyped participants included in the PRS study mapped against the 1,000 Genome European population.

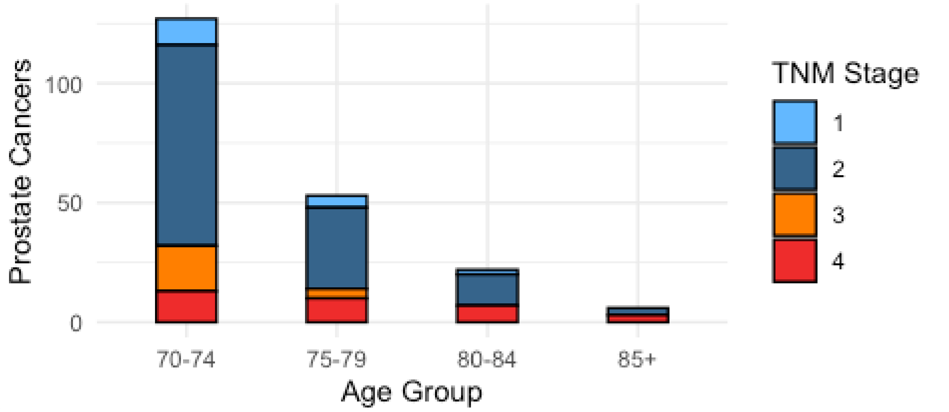

Figure S2. TNM staging by age. Staging information identifying the TNM stage at which the tumour was identified, stratified by the age of incident prostate cancer.

Table S1. Association of the PRS with prevalent prostate cancer.

**Self-reported prostate cancer** *(658 pre-trial self-reported cases)*

	PRS as Continuous Variable			PRS as Categorical Variable		
	**Odds Ratio**	**95% CI**	**p-value**	**Odds Ratio**	**95% CI**	**p-value**
**Family History***	2.71	(2.15; 3.39)	<0.0001	2.73	(2.17; 3.41)	<0.0001
**PRS** (per std dev)	1.80	(1.65 ; 1.96)	<0.0001			
**Low PRS**<br>0-20% (N=62)				Reference		
**Medium PRS**<br>20-80% (N=346)				1.93	(1.47 ; 2.57)	<0.0001
**High PRS**<br>80-100% (N=250)				4.63	(3.48; 6.26)	<0.0001
